# Non-natural ruthenium isotope ratios of the undeclared 2017 atmospheric release consistent with civilian nuclear activities

**DOI:** 10.1038/s41467-020-16316-3

**Published:** 2020-06-09

**Authors:** Timo Hopp, Dorian Zok, Thorsten Kleine, Georg Steinhauser

**Affiliations:** 1 Institut für Planetologie, University of Münster, Wilhelm-Klemm-Str. 10, 48149 Münster, Germany; 2 Institute of Radioecology and Radiation Protection, Leibniz Universität Hannover, Herrenhäuser Str. 2, 30419 Hannover, Germany; 3 Present Address: Origins Laboratory, Department of the Geophysical Sciences and Enrico Fermi Institute, The University of Chicago, Chicago, IL 60637 USA

**Keywords:** Atmospheric chemistry, Environmental impact, Atmospheric chemistry

## Abstract

Understanding the circumstances of the undeclared 2017 nuclear release of ruthenium that led to widespread detections of the radioisotope ^106^Ru in the Eurasian region, and whether it derives from a civilian or military source, is of major importance for society and future improvements in nuclear safety. Until now, the released nuclear material has merely been studied by analyzing short-lived radioisotopes. Here, we report precise measurements of the stable isotopic composition of ruthenium captured in air filters before, during, and after the nuclear release, and find that the ruthenium collected during the period of the 2017 nuclear release has a non-natural isotopic composition. By comparing our results with ruthenium isotopic compositions of spent nuclear fuels, we show that the release is consistent with the isotopic fingerprints of a civilian Russian water-water energetic reactor (VVER) fuel at the end of its lifetime, and is not related to the production of plutonium for nuclear weapons.

## Abstract (web summary)

A cloud of enhanced ruthenium concentrations has been observed over Europe in 2017, but no country has acknowledged responsibility for this nuclear release. Here, the authors show that the stable isotopic composition of ruthenium emitted from nuclear fuel reprocessing during the 2017 event is consistent with the isotopic signature of civilian Russian nuclear reactor fuel.

## Introduction

A nuclear accident may become a serious hazard for humankind and exhibit long-lasting consequences for the environment. Decades ago, and especially in the aftermath of the Chernobyl nuclear accident, global networks of monitoring stations were established for atmospheric radioactivity surveillance. They now have the sensitivity and precision to identify atmospheric releases of even small amounts of anthropogenic radionuclides^1^. In September and October 2017, these monitoring stations detected a radioactive cloud over a wide swath of Europe containing the fission products ^106^Ru (*T*_1/2_ = 371.8 d) and traces of ^103^Ru (*T*_1/2_ = 39.2 d)^2^. The characteristics of the release (e.g., lack of concomitant radionuclides) suggested that the source was a spent nuclear fuel reprocessing facility. The source term of the release was estimated at 250 TBq ^106^Ru, and atmospheric modeling indicated that the cloud originated in the southern Urals in the Russian Federation^2,3^. This area hosts one of the largest nuclear facilities in the world, the Federal State Unitary Enterprise (FSUE) Production Association Mayak in Ozersk, Russia.

Currently, no country has assumed responsibility for this considerable release, which is likely the single-largest accidental release from civilian nuclear reprocessing^4^. Despite a large number of meteorological indications^3,5–8^, Russian authorities and institutions have repeatedly denied any involvement of the Mayak facility in the release^9–11^. In their official statement^9^, the Rosatom State Nuclear Energy Corporation emphasized that there were not any incidents at any of the Rosatom sites during the period of September–October 2017. The FSUE Production Association Mayak is a subsidiary of Rosatom. The Russian authority also referred to this statement in response to a query concerning the release of ^106^Ru from the International Atomic Energy Agency (IAEA) to its 43 member states in the region^12^. According to IAEA, none of the countries reported an event that could be the cause of the release of ^106^Ru in the fall of 2017^12^. In an interview with Nuclear Engineering International Magazine, the deputy director of the Nuclear Safety Institute of the Russian Academy of Sciences (IBRAE) argued that “if the Mayak facility [were] the source, then we would have found concentrations hundreds of thousands of times the norm around it and in the soil^10^.” The IBRAE also set up an International Independent Scientific Commission for the investigation of the release of ^106^Ru. The Commission gathered two times, in January and April 2018^13,14^. The Commission agreed on an estimated release source term of the event of ~100 TBq^13^. Science reported that a representative of the Russian nuclear regulator Rostechnadzor who inspected Mayak in November 2017 told the Commission that he saw no anomalies in the Mayak facility from a month earlier^11^. Early alternative attempts at explanation of the release, such as a release on Romanian territory^9^ or the burning of a satellite’s radionuclide battery containing ^106^Ru^10^ had been addressed previously^2^ and were essentially dispelled. While it is difficult to imagine that a private facility could routinely handle such considerable activities, it is clear that nuclear facilities (both private and state-run), including reprocessing facilities, must be operated under strict governmental regulatory control^15^ and report any events to the regulator.

Previous studies have focused on tracking the cloud across Europe and have provided chemical insights, suggesting that the release occurred at an advanced stage of the reprocessing, when the Ru species had been transformed from initially produced gaseous RuO_4_, at least in part, into one or more soluble compounds with medium volatility^2^. One of the released chemical species was identified as a polychlorinated Ru(III) form^16^. The release carried a ^103^Ru/^106^Ru signature of very young spent fuel (i.e., only 1.5 or 2 years after the end of neutron irradiation, assuming regular high-burnup fuel)^2^. Together with other indications, this suggests that the ^106^Ru release could have originated during the production of a highly radioactive ^144^Ce source commissioned for the European sterile neutrino project SOX-Borexino in the Gran Sasso National Laboratory (GSNL)^2,11^.

The degree of burnup of the reprocessed fuel is key for understanding the fuel’s past use prior to the release. High burnup would imply a civilian purpose of the spent fuel. Low burnup, by contrast, may indicate a military purpose, such as production of weapons-grade Pu or even utilization of low-burnup fissile material in a nuclear-powered missile^17^. With increasing burnup, nuclear fuel will increasingly accumulate ^240^Pu, which thwarts its applicability in nuclear warheads.

Any use of low-burnup fuel would also affect the model age of the released material. The above model age of 1.5–2 years after neutron irradiation applies only to high-burnup fuel. In particular, if low-burnup fuel had been used to produce the ^144^Ce source above mentioned, the measured ratio of ^103^Ru/^106^Ru would make the released material appear younger than its actual age. In other words, low burnup could also mean that the fuel that was used for the ^144^Ce source was in fact “older” than the suggested ≤2 years. This could mean that it underwent the established and safe reprocessing scheme with ~3 years of cooling. As outlined in ref. ^18^, the compact design of the ^144^Ce source required exceptionally high specific activity, which is only achievable either by reducing the minimum cooling time from 3 to 2 years (high-burnup scenario) or by reprocessing fuel that has undergone only approximately one-third of its nominal burnup (i.e., prior to reaching the end of its lifetime), while allowing 3 years of cooling (low-burnup scenario). In any case, since Mayak not only hosts a reprocessing facility but also has an explicit military history, it has until now not been possible to rule out a military context or another low-burnup scenario of the release.

The circumstances of the incident cannot be assessed solely by analyzing the detected radioactive Ru isotopes, because the resulting ^103^Ru/^106^Ru ratio is a function of neutron flux, energy spectrum, fuel type that varies by reactor type and burnup, and decay time. Since none of these variables are known, the ^103^Ru/^106^Ru ratios do not allow the direct distinction of the provenance of the released material. The stable isotopic composition of fission-generated Ru also depends on the fuel type, hence, varies by reactor type and burnup, but not on radioactive decay. Therefore, precise measurements of the stable isotopic composition of fission-generated Ru can serve as an indicator of whether the released material was produced in a civilian reactor or during a low-burnup scenario, e.g., the production of weapons-grade Pu^19–21^.

Here, we show that precise stable isotope analyses of Ru in environmental samples can be used to constrain the provenance of nuclear material released into the atmosphere. We present the first high-precision measurements of stable Ru isotopic compositions of particulate matter collected in air filters including one sample that contains material from the 2017 Ru release. We conclude that the stable isotopic composition of the 2017 nuclear release is consistent with fission-generated Ru produced in regular, high-burnup spent fuel; hence, the nuclear release was most likely related to an accident during reprocessing of spent fuel used in civilian nuclear activities.

## Results

### Ruthenium isotopic compositions of air filter samples

**Fig. 1:**
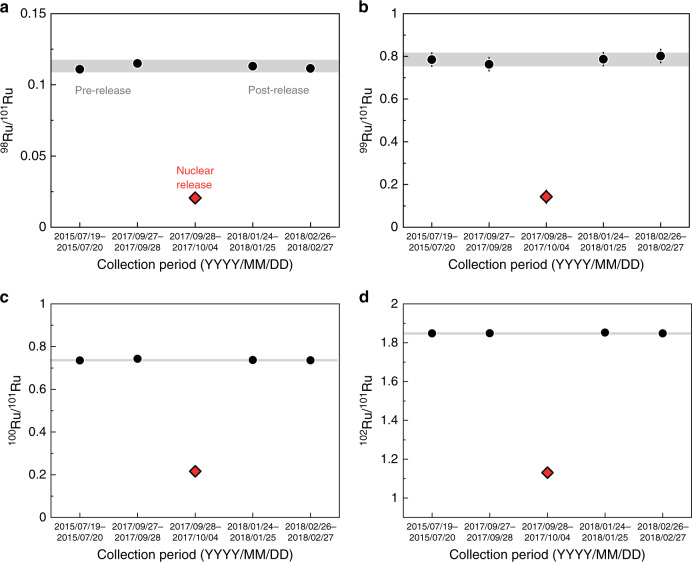
Isotope ratios from a series of air filters from Vienna, Austria. Isotope ratios for **a** ^98^Ru/^101^Ru, **b** ^99^Ru/^101^Ru, **c** ^100^Ru/^101^Ru, and **d** ^102^Ru/^101^Ru. The ^106^Ru containing sample air filter of the 2017 atmospheric release of nuclear Ru (red diamond) exhibits distinctly non-natural Ru isotope ratios. Four reference air filter samples (black circles) collected from 2015 to 2018 have similar Ru isotope ratios that agree with natural Ru and are indicative of the typical background Ru collected by air filters in Vienna. The gray bars represent the standard deviation (2 s.d.) on the average isotope ratios of the four reference filters and are treated as the external reproducibility of the measurements. Error bars on individual samples correspond to 2 s.e. (*n* < 3) or 2 s.d. (*n* ≥ 3) of measurements and are, if not visible, smaller than the symbols.

Isotopic anomalies caused by the decay of anthropogenic radioisotopes are extremely difficult to observe in regular environmental samples as the additional input of the decay products will be usually insignificant compared with the overwhelming abundance of natural occurring isotopes. Consequently, such anomalies have been reported only for environments with a high level of radioisotope contamination, i.e., those that were directly impacted by anthropogenic nuclear activities^22,23^. However, alongside the radioisotopes, almost any nuclear release also contains stable isotopes of the same element, but with anomalous, non-natural abundances that reflect production by nuclear fission and capture reactions. We investigated the stable isotopic compositions of a few nanograms of airborne Ru collected by a series of air filters from Vienna, Austria, including one sample that incorporated radioactive Ru from the atmospheric release in 2017^24^ using multicollector inductively coupled plasma mass spectrometry (see Methods). Four other filters from air collections between 2015 and 2018 (Supplementary Table 1) are used to characterize the typical background Ru isotopic composition of particulate matter sampled at that specific air filter station (Fig. 1; Supplementary Notes 1 and 2; Supplementary Tables 2 and 3). Two of these filters were used to collect particulate matter prior to the 2017 nuclear release; the other two were in operation after the radioruthenium episode. Our results show that all four background air filters exhibit isotope ratios of ^98^Ru/^101^Ru, ^99^Ru/^101^Ru, ^100^Ru/^101^Ru, and ^102^Ru/^101^Ru in excellent agreement with one another and with natural Ru isotope abundances (Fig. 1). By contrast, the sample filter that collected air at the same station during the time of the radioruthenium episode over Europe in September/October 2017 (2017/09/28 to 2017/10/04) displays drastically different, non-natural Ru isotopic composition compared with the defined background of environmental (terrestrial and potentially anthropogenic) Ru (Fig. 1). The significant shift of the ^i^Ru/^101^Ru ratios is on the order of tens of percent. Natural processes cannot produce isotope fractionations of such magnitude for heavy elements^25–27^.

### Isotope abundances of natural and fission-derived ruthenium

**Fig. 2:**
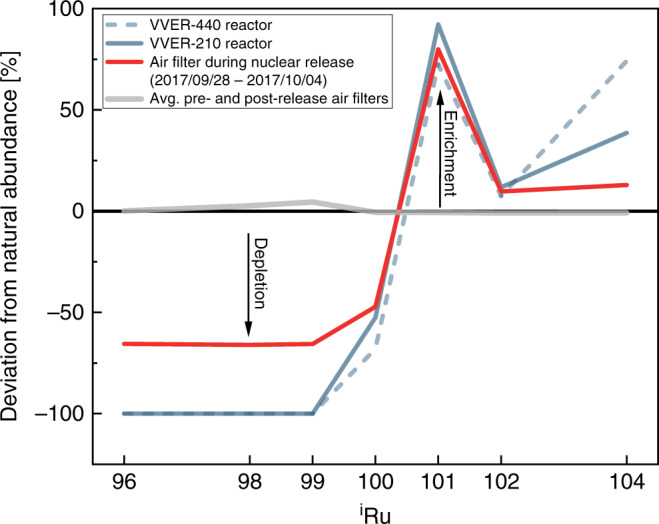
Ruthenium isotope abundances of the radioactive air filter and Ru produced by nuclear reactors relative to natural Ru. Ruthenium produced by nuclear reactors, e.g., VVER-type reactors (blue lines), contain negligible amounts of ^96^Ru, ^98^Ru, and ^99^Ru, whereas heavier Ru isotopes (^101^Ru, ^102^Ru, and ^104^Ru) are enriched relative to natural Ru^27,28^. The gray line gives the abundances calculated for the reference filters from 2015 to 2018. The isotope abundances derived for the non-natural Ru in the air filter (red line) display the typical depletion of the lighter and enrichment of the heavier isotopes produced by mixing of nuclear reactor-derived Ru with natural Ru.

A key observation from our data is that, compared with natural Ru^28^, the Ru stable isotopic composition of the air filter from the radioruthenium episode is depleted in the lighter Ru isotopes (^96^Ru, ^98^Ru, ^99^Ru, and ^100^Ru), while the heavier Ru isotopes (^101^Ru, ^102^Ru, and ^104^Ru) are enriched. This pattern is consistent with the expected composition of Ru produced during nuclear fission, because the formation of ^96^Ru, ^98^Ru, ^99^Ru, and ^100^Ru is suppressed by stable Mo and long-lived Tc nuclides along the beta-decay series of these isobars. By contrast, the formation of ^101^Ru, ^102^Ru, and ^104^Ru is not inhibited by other isotopes, and they are the stable termini of their beta-decay series (Fig. 2). The non-natural Ru isotopic composition of Ru collected during the radioruthenium episode, therefore, indicates a significant contribution of fission-generated Ru to the particulate matter collected in the investigated air filter.

## Discussion

**Fig. 3:**
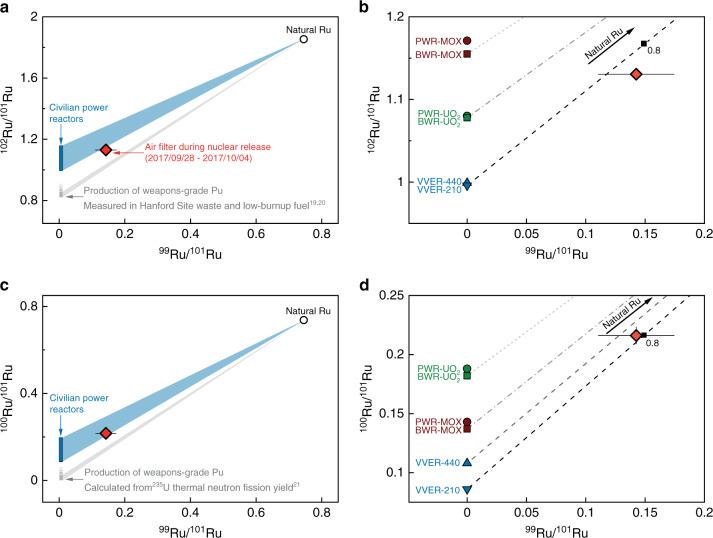
Source identification of the nuclear Ru released into the atmosphere based on Ru isotope ratio plots. Subplots (**a**) and (**b**) show ratios of ^102^Ru/^101^Ru versus ^99^Ru/^101^Ru and (**c**) and (**d**) show ^100^Ru/^101^Ru versus ^99^Ru/^101^Ru. Ruthenium produced in civilian power reactors (blue rectangle)^29^ has distinctive isotope compositions compared with Ru produced during the production of weapons-grade Pu (gray rectangles) (**a**, **c**) (e.g., Hanford Site^19^, low-burnup fuels^20^, or ^235^U thermal neutron fission yield^21^). A higher contribution of ^239^Pu fission may increase the ^102^Ru/^101^Ru and ^100^Ru/^101^Ru ratios. The Ru isotopic composition of the sample air filter falls within mixing arrays of Ru produced in civilian power reactors and natural Ru (blue fields) (**a**, **c**). Different types of power reactor and nuclear fuels have distinctive Ru isotope compositions: Pressurized water reactor (PWR)—circle; boiling water reactor (BWR)—square; mixed oxide fuel (MOX)—brown; uranium oxide fuel (UO_2_)—green; water–water energetic reactor (VVER-210 or VVER-440)—blue (**b**, **d**)^29^. Less common reactor types such as pressurized heavy-water reactors or low-power reactors such as research reactors were not considered here. The atmospheric Ru released in 2017 falls on mixing lines with VVER-type nuclear fuel and natural Ru with ~65–90% of the total Ru derived from nuclear fuel (**b**, **d**). Error bars correspond to the external reproducibility (2 s.d.) of the four reference filters (Supplementary Table 3).

Most importantly, our results can be used to assess the provenance of the non-natural Ru. This is because ^235^U and ^239^Pu fission are characterized by distinct thermal fission yields of Ru isotopes, where ^239^Pu fission favors heavier Ru isotopes compared with ^235^U fission. Weapons-grade Pu is produced from low-burnup uranium fuel to minimize the ingrowth of ^240^Pu during irradiation and contains a fission product signature consistent with ^235^U fission. In contrast, civilian power production reactors use a very high-burnup fuel cycle that not only produces a much higher ^240^Pu content, but also a significantly higher overall Pu content. As a result, at the end of the nuclear fuel’s lifetime, over 50% of fissions in civilian reactors come from ^239^Pu bred in during irradiation, producing a different fission product signature that is a mix of ^235^U and ^239^Pu fission yields. These differences explain why fission-generated Ru has distinct isotopic compositions when produced in civilian power reactors versus low-burnup operation for the production of weapons-grade Pu or other low-burnup scenarios (Fig. 3; Supplementary Note 3; Supplementary Table 4). Specifically, Ru produced in civilian power reactors has higher ^100^Ru/^101^Ru and ^102^Ru/^101^Ru ratios due to the increasing contribution of ^239^Pu fission in the later lifetime of energy-producing nuclear fuel (Fig. 3)^29^. By contrast, the ^100^Ru/^101^Ru and ^102^Ru/^101^Ru ratios measured from the (military) Hanford Site groundwater are much lower, due to ^235^U fission dominating the signal with only marginal input from ^239^Pu fission (Fig. 3a, c).

Given that the air filters before and after the ^106^Ru release already contained natural ruthenium, the Ru collected during the radioruthenium episode is expected to be a mixture between fission-generated Ru and natural ruthenium (Supplementary Note 4). Accordingly, the Ru isotope ratios of the air filter containing the fission-generated Ru should fall on a mixing array of natural Ru with the characteristic signatures of the source of the fission-generated Ru. Consistent with this, the Ru stable isotopic composition from the radioruthenium episode air filter falls on mixing arrays with civilian power reactors, but not on mixing arrays with Ru generated during low-burnup scenarios such as ^239^Pu production (Fig. 3a, c). Hence, the atmospheric release of fission-generated Ru was most likely related to spent nuclear fuel used in civilian power reactors, whereas an origin related to weapons-related Pu production or other low-burnup scenarios is less likely. Nuclear fuel used in different types of civilian nuclear reactors has varying contents of ^235^U and ^239^Pu. For instance, mixed oxide fuel (MOX) uses both ^235^U and ^239^Pu, typically from spent fuel reprocessing, whereas most other reactor fuel is based on low enriched ^235^U (Supplementary Tables 5 and 6). Therefore, MOX carries a distinct Ru isotopic signature compared with other fuel types with lower ^239^Pu contents (Fig. 3b). This means that Ru isotopic signatures can help not only distinguishing a civilian from a military origin of anthropogenic Ru, but also to differentiate between different types of the civilian power reactors. For instance, the isotopic characteristics of various common civilian power reactor types, in particular Western pressurized water reactors (PWR), boiling water reactors (BWR), and water–water energetic reactor types (VVER) (a Russian version of PWR) are distinct^29^ (Fig. 3b, c). The VVER-210 is the discontinued lower-power version of the VVER family, whereas the VVER-440 is still frequently used (Supplementary Note 5). The Ru isotopic signature of the sample filter is distinct from the expected composition of Ru from nuclear power reactors using MOX or uranium oxide fuel mixed with natural Ru (Fig. 3b, d). In contrast, the sample filter Ru falls on a mixing line of natural Ru and anthropogenic Ru produced in VVER reactors for both ^102^Ru/^101^Ru and ^100^Ru/^101^Ru, respectively (Fig. 3b, d; Supplementary Note 4; Supplementary Table 7). Mass balance calculations predict that around 65–90% of the total Ru found in the sample filter in question is attributable to a reactor (Fig. 3b, d). Furthermore, the isotope abundances of the VVER reactor types and the air filter compared with the natural Ru isotope abundances confirm that the Ru in the air filter is a mixture of natural Ru with fission-generated Ru most likely produced in a VVER reactor. Such a mixture has the typical depletion in ^96^Ru, ^98^Ru, ^99^Ru, and ^100^Ru and enrichment in ^101^Ru, ^102^Ru, and ^104^Ru (Fig. 2). Thus, our data are best explained by a scenario in which the undeclared radioactive Ru release of September 2017 carried stable isotope ratios that match the signature of spent VVER reactor-type nuclear fuel at the end of its lifetime, mixed with a minor component of natural Ru.

The Russian Federation operates five reactors of the VVER-440 type, one of which is located at Novovoronezh NPP and four at Kola NPP (Supplementary Note 5; Supplementary Table 8). Kola NPP was en route of a railway transportation (Supplementary Fig. 1) of naval spent nuclear fuel from Andreeva Bay^30^ via Murmansk that arrived in Mayak 6 weeks prior to the estimated release date^31^. Since there is both a temporal and spatial coincidence, this fuel shipment may potentially be relevant to the release. In any case, while pressurized water reactors of the VVER type are commonly used in many countries, there are comparatively very few operating reprocessing plants. Consistent with the recent reconstruction and air modeling of the origin of the radioruthenium cloud,^2,3,5^ the Ru stable isotope signature points to the involvement of a reprocessing facility. Furthermore, the fission-generated Ru component in the air filter displays the signature of spent VVER nuclear fuel. This result is important in the context of a possible connection between the nuclear release and the production of a compact ^144^Ce source for the Borexino antineutrino detector at the GSNL. While it would have been possible to meet the specifications of the ^144^Ce source by using spent fuel with low burnup^2^, the Ru stable isotope signature confirms that spent fuel at the end of its lifetime was reprocessed^32–34^. In this case, the cooling time of the nuclear fuel must have been decreased to ≤2 years to achieve the required high specific activity of ^144^Ce in the compact CeO_2_ source^18,33^. Hence, the combination of atmospheric observations of the undeclared nuclear release and the Ru stable isotope signature of the released Ru suggests that the release may have occurred during reprocessing of regular spent VVER-type fuel after a shorter than typical cooling time, most likely at the Mayak reprocessing plant. This is the first observation of nuclear-related Ru stable isotope signatures in air particulate samples and a demonstration of its forensic value for assessing the provenance of the release. The correlation of the air filter’s isotopic signature with spent VVER-440 fuel is consistent with the fact that spent VVER-440 fuel is routinely reprocessed at the Mayak facility, whereas fuel from VVER-1000 or from RBMK power reactors is not^35^ (Supplementary Note 5).

## Methods

### Air filter samples

The air filters used in this study are square polypropylene (PP) filters with rectangular shape (size 46 × 57 cm). They are employed in a high-volume air filter station (675 m³·h^−1^) on the rooftop (~100 m above ground) of a building in Vienna, Austria (48.23 N; 16.42 E)^24^. Air sampling is performed at this station with an air collection time of usually 1 day or up to 1 week. After sampling, the filter is removed from the sampler and pressed into a round shape with a diameter of 5 cm. This geometry is preferred for routine gamma measurements. Five air filters from the years 2015 to 2018 were investigated in this study (Supplementary Table 1). Within this sample set, two filters prior and two after the incident (Supplementary Table 1) bracket the air filter that collected radioruthenium during the week from 2017/09/28 to 2017/10/04. Note that one sample was taken the day before the radioruthenium episode over Austria (2017/09/27). Hence, the set of samples allows comparing the isotopic composition of the regular, environmental Ru background at the air filter station with the isotopic composition of the fission-generated (and radioactive) Ru-impregnated air filter. In addition, a pristine (blank) filter that was doped with ~20 ng Ru from an Alfa Aesar^TM^ standard solution was used to test the sample preparation and chemical separation, as well as to estimate the reproducibility of the MC-ICPMS measurements (Supplementary Table 1).

### Chemicals and standard solution

The sample preparation was performed at the Institute of Radioecology and Radiation Protection at the Leibniz University Hannover. For digestion, nitric acid with Millipore Emsure^TM^ grade and hydrochloric acid with Millipore Suprapur^TM^ grade from Merck were used. Dilutions with water were done with Merck Millipore Milli-Q^TM^ water (18.2 MΩ cm). The ashing was performed in porcelain crucibles from Morgan Advanced Materials Haldenwanger. Cellulose filter with particle retention of lower than 2 µm were purchased from GE Healthcare - Whatman^TM^. The chemical purification of Ru was performed in a class-10,000 clean room environment using class-10 laminar flow hoods in the Institut für Planetologie at the University of Münster. We used pre-cleaned Savillex Teflon perfluoralkoxy (PFA) vials and bottles. Acids (HNO_3_ and HCl) of Millipore Emsure^TM^ grade were double distilled in Savillex^TM^ DST-1000 Acid Purification Systems. Dilution of chemicals was conducted with Merck Millipore Milli-Q^TM^ water (18.2 MΩ cm). In the absence of a certified standard reference solution for Ru, we used an in-house Ru standard solution purchased from Alfa Aesar.

### Sample preparation for MC-ICPMS analyses

The filters were cut in ~2.5 g pieces and ashed in an oven over night at 450 °C. The ashed samples were transferred four times with 2.5 ml of 69% nitric acid into a 110 mL polytetrafluoroethylene (PTFE) vessel. The sealed vessels were put into a MARS 6 microwave digestion system from CEM Corporation^TM^ for a more efficient digestion. These were heated 20 min to a temperature of 160 °C and held at this temperature for 30 min. After cooling, the solutions were filtered through a cellulose filter with a particle retention of 2 µm. The filtrates were evaporated to dryness and dissolved in 5 ml of 6 M hydrochloric acid. Due to the matrix change, a second filtration step was performed to yield a particle-free solution.

The chemical separation of Ru followed a modified two-stage ion exchange chromatography procedure based on the method outlined in ref. ^36^. After filtration, the sample solutions were transferred into 15 ml Savillex PFA beakers and then dried down at 100 °C on a hot plate. Cations were converted into their chloride form using multiple dry-downs in 6 M HCl and re-dissolved in 5 ml of 0.2 M HCl. In the first step, the sample solutions were loaded onto cation exchange columns filled with 10 mL pre-cleaned BioRad AG 50W-X8 (100–200 mesh) resin. On these columns, the bulk of the Ru was eluted in a total volume of 14 mL 0.2 M HCl, while the major elements (i.e., Fe and Ni) remain adsorbed on the resin. Then the Ru fractions were dried down on a hot plate and re-dissolved three times using 5 mL of 1 M HF. To remove remaining interfering elements (Zr, Mo, Pd), the Ru fractions were dissolved in 7 mL 1 M HF and were loaded onto anion exchange columns filled with 2 ml of pre-cleaned BioRad AG 1-X8 (100–200 mesh) resin. Ruthenium was eluted in 14 mL 1 M HF, whereas Zr, Mo, and Pd remain adsorbed onto the resin. The final Ru fractions were dried and re-dissolved in 0.5 mL 0.28 M HNO_3_.

This procedure led to Ru fractions of the air filter samples with Mo/Ru < 0.03 and Pd/Ru < 0.009 (for the non-natural Ru filter <0.003 and <0.001, respectively) that allow for precise interference correction on ^96^Ru, ^98^Ru, ^100^Ru, ^102^Ru, and ^104^Ru within the precision given on the Ru isotope data. The total amount of Ru available for isotope ratio measurements by MC-ICPMS were determined by comparing the intensity of a ~10% aliquot of each sample solution to a standard. The amounts of Ru available ranged between 0.4 to 3.4 ng.

### Mass spectrometry and data reduction

The Ru isotope measurements were conducted using a Thermo Scientific Neptune Plus MC-ICPMS at the Institut für Planetologie in Münster, Germany. Prior to the measurements, samples were dissolved in 0.28 M HNO_3_ and were introduced into the mass spectrometer using a CETAC Aridus II desolvating system combined with an 80 µL·min^−1^ ESI PFA nebulizer. The formation of oxides was monitored as CeO/Ce and reduced to <1% by the addition of N_2_ to the sample gas. Sample and standard solutions were measured at concentrations of ~10 and ~1 ppb using conventional Ni H cones. Ion beams were simultaneously collected in static mode for all seven stable Ru isotopes (^96^Ru, ^98^Ru, ^99^Ru, ^100^Ru, ^101^Ru, ^102^Ru, ^104^Ru) together with ^97^Mo and ^105^Pd as interference monitors. Ion beams were measured using Faraday cups connected to 10^11^ Ω feedback resistors, except the ion beams of ^99^Ru and ^101^Ru that were collected using 10^12^ Ω feedback resistors. Total ion-beam intensities corresponded to ~110 V·ppm^−1^. Sample measurements comprised 30 × 4.2 s integrations of the ion beams and consumed ~2 ng Ru for 10 ppb and ~0.2 ppb Ru for 1 ppb solutions, respectively. The baselines were measured on peak with 40 × 8.4 s integrations on a solution blank prior each measurement. Each sample measurement was bracketed by measurements of an Alfa Aesar Ru standard solution with matching concentration (±10%). After each sample or standard measurement, the system was rinsed for 4 min (1 ppb Ru solution) or 8 min (10 ppb Ru solution), respectively.

### Accuracy and reproducibility

The measured Ru isotope ratios of the air filter samples were corrected for instrumental mass bias by normalizing the bracketing standards to ^99^Ru/^101^Ru of 0.745 following the exponential law and using the average isotope fractionation factor (*β*) calculated from the two bracketing standards. Interferences of Mo and Pd on ^96^Ru, ^98^Ru, ^100^Ru, ^102^Ru, and ^104^Ru were corrected with the mass bias corrected ^97^Mo and ^105^Pd monitors.

Supplementary Table 2 shows the long-term reproducibility of 10 and 1 ppb standard solution measurements (including all bracketing standard measurements). In addition, a Ru-doped (~20 ng) blank filter was processed through the complete chemical purification procedure to determine the external reproducibility. The 10 and 1 ppb measurements of this Ru-doped blank filter show a standard deviation of ~0.002 (2 s.d.) on all Ru isotope ratios (Supplementary Table 2). Of note, the deviation on ^102^Ru/^101^Ru of the 10 ppb Ru-doped blank filter solution relative to the standard (i.e., natural ^102^Ru/^101^Ru) can be explained by a higher Pd/Ru ratio (>0.01) possibly due to contamination of the 10 ppb solution prior measurements. The Ru isotope ratios determined from the 1 ppb Ru-doped filter measurements (Pd/Ru < 0.008) agree within uncertainty with standard solution measurements (Supplementary Table 2; Supplementary Fig. 2). All other air filter samples analyzed in this study have Pd/Ru < 0.008 and Mo/Ru < 0.03. The air filter containing the fission-generated and radioactive Ru has Pd/Ru < 0.001 and Mo/Ru < 0.003.

## Supplementary information

Supplementary Information

## Data Availability

No custom code or mathematical algorithm was used in this study.

## Data availability

All data are available in the main text or in the Supplementary Materials.
