# Supplementary material for: Non-natural ruthenium isotope ratios of the undeclared 2017 atmospheric release consistent with civilian nuclear activities
Source: Nat Commun. 2020 Jun 9;11:2744. doi: 10.1038/s41467-020-16316-3 (PMC7283356; doi:10.1038/s41467-020-16316-3)
Supplement: Supplementary file 1 — Supplementary Information [file 41467_2020_16316_MOESM1_ESM.pdf]

Supplementary Information

Non-natural ruthenium isotope ratios of the undeclared 2017 atmospheric release consistent with civilian nuclear activities

Hopp & Zok *et al.*

Supplementary Notes

Supplementary Note 1. Analyses

To estimate the true external reproducibility of used air filter samples, standard deviation (2 s.d.) of the Ru isotope ratios determined for the reference air filters can be calculated (Supplementary Table 3). This more conservative uncertainty estimate using the standard deviation (2 s.d.) on the Ru isotope ratios of all four reference air filters may include some natural variations of the Ru isotopic composition in the atmospheric Ru over time (i.e., atmospheric Ru may partly originate from vehicle catalytic converters that may have led to isotope fractionation). However, the overall good agreement of all four filter samples collected from 2015 to 2018 can be used to describe and characterize the general isotopic composition of "background Ru" in the atmosphere over Vienna, Austria (Supplementary Table 3). Measurements of 10 ppb and 1 ppb solutions of the standard solution and several of the air filters show good reproducibility and accuracy of the measurements and no systematic differences due to low amount of Ru analyzed by 1 ppb solution measurements (Supplementary Figure 2; Supplementary Tables 2 and 3).

The air filter containing the non-natural Ru from the nuclear release in 2017 could also be analyzed twice, using 10 ppb and 1 ppb solutions, respectively. The two measurements agree very well with each other and reveal highly non-natural Ru isotopic composition of the Ru collected by this air filter for all Ru isotope ratios (Supplementary Table 3).

Supplementary Note 2. Isotopic composition of fission-generated Ru

Supplementary Table 4 summarizes the Ru isotopic composition of fission-generated Ru from different reactor types and the thermal neutron fission yields expected for low-burnup ^{239}Pu production. These values were derived from actual measurements of nuclear fuels, nuclear waste, contaminated ground water, or calculated based on fission product yields[1-4]. Fission-

generated Ru does not have significant abundances of ^{96}Ru, ^{98}Ru, and ^{99}Ru, because long-lived or stable molybdenum and technetium isotopes inhibit the beta-decay along the corresponding isobars, respectively. Artificial generation of these lighter Ru isotopes are possible at trace levels only due to (rather improbable) independent fission events yielding these Ru isotopes directly (or by the extremely slow decay of ^{99}Tc, respectively). In contrast, the heavier Ru isotopes (^{101}Ru, ^{102}Ru, and ^{104}Ru) are produced in higher abundances than the natural abundances because their production path along the isobar is not blocked by any long-lived or stable isotope. The isotopic deviation of fission-generated Ru vs. naturally abundant Ru is greatest for the isotope ^{101}Ru (Supplementary Table 4). Hence, the ^{i}Ru/^{101}Ru ratios can be used to distinguish between various origins of fission-generated Ru and thus were favored for the illustration of the nuclear impact on the isotopic Ru composition in Fig. 1.

Available data of ^{102}Ru/^{101}Ru in low-burnup nuclear fuel[2] and the Hanford Site groundwater[1] allow deriving an estimate for the expected Ru isotopic composition generated during the production of weapons-grade Pu (Supplementary Table 4). This estimate is in good agreement with the calculated ^{102}Ru/^{101}Ru of ^{235}U thermal neutron fission yields, with minor contributions of ^{239}Pu thermal neutron fission (Supplementary Table 4). The estimated ^{102}Ru/^{101}Ru ratio for Ru produced during ^{239}Pu production is different from any of the ^{102}Ru/^{101}Ru signatures of civilian reactors (Supplementary Table 4). Moreover, during low-burnup and thermal neutron fission of ^{235}U or ^{239}Pu, no significant amount of ^{100}Ru is produced, hence, the ^{100}Ru/^{101}Ru ratio of Ru from civilian reactors is also a distinctive signature for tracing the origin of fission-generated Ru (Supplementary Table 4).

Supplementary Note 3. Ru isotopic fingerprints of various reactor types

In preparation of the German nuclear waste repository, the ‘Gesellschaft für Anlagen- und Reaktorsicherheit’ (GRS)[4] modelled and published the isotopic inventory of all spent nuclear fuel from all reactor types that have ever been in operation in Germany. The link

https://www.grs.de/en/node/1749 will bring you to the root menu, from where the tabulated data can be found under “Anhang GRS-278.” For this study, we used “Waste from the utilization of power reactors” (1 Abfälle aus der Nutzung von Leistungsreaktoren), “Irradiated nuclear fuel elements” (Bestrahlte Brennelemente), “Nuclear fuel” (Kernbrennstoff). The reactor types used for this study are “Pressurized water reactors” with both MOX and UO_2 as fuel (DWR-MOX and DWR-UO2, respectively), Boiling water reactors” (SWR-MOX and SWR-UO2, respectively), VVER-210 (KKR), and VVER-440 (KGR).

The enrichment and burnup parameters used in the GRS modelling are tabulated in Supplementary Table 5. Chemical impurities are listed in Supplementary Table 6. Fuel cladding was Zircaloy 4. Nuclear cross sections were taken from the library ENDF/B VI.

Supplementary Note 4. Estimate of non-natural Ru fraction

The non-natural Ru isotopic composition in the air filter can be explained by mixing of fission-generated Ru from the undeclared atmospheric release in 2017 with natural Ru. The determined isotopic composition falls closest to mixing lines of the Ru expected to be produced by a VVER reactor and natural Ru. The fraction of fission-generated Ru required can be estimated by mass-balance calculations using the calculated Ru isotope abundances (Supplementary Table 7) and is, depending on the Ru isotope used in the calculation, ~66-90 % (excluding ^{104}Ru due to possible larger interference effects from ^{104}Pd). This means that 10 to 34 % of the Ru analyzed in the sample solution was natural atmospheric Ru collected from the air or added as analytical blank during digestion and chemical separation (despite using ultra-high-purity chemicals). In any case, the majority of the Ru analyzed in this filter sample was generated in a nuclear reactor. To better estimate the absolute amount of Ru that was collected by the air filter station in Vienna, Austria, during the week of the 2017/09/28 to 2017/10/04, we analyzed a small aliquot (2 %) of the sample solution for its Ru concentration using a ThermoScientific *X-Series 2* quadrupole ICPMS at the Institut für Planetologie in Münster. The measured intensity of the

sample solution was compared to a standard solution to estimate the total amount of Ru in the sample aliquot. To calculate a true estimate of the Ru content, the non-natural isotopic composition of the Ru in the air filter has to be taken into account (Supplementary Table 7). After correction for the non-natural isotopic composition, the amount of Ru in the sample solution is estimated to be ~5 ng (±20 %). Hence, during chemical separation and removal of Mo and Pd interferences, up to 50 % of the Ru was lost. If we assume that ~66 to 90 % of the total Ru trapped on the filter is fission-generated, it translates to 3-4.5 ng in the ~2.5 g filter material digested (Supplementary Table 1). Since the fission-generated Ru was homogeneously distributed (as shown in [5]), we can estimate the total amount of fission-generated Ru collected in the complete filter (total mass 9.7 g) to be 11.6-17.5 ng. The total amount of Ru released into the atmosphere during the undeclared release is not well known, but can be estimated to ~110 g, based on the Ru isotopic composition of spent fuel and the ^{106}Ru source term of 250 TBq. In combination with these estimations, a fraction of ~1x10^{-10} of the total released stable Ru and ~8 x10^{-12} of the radioactive Ru was collected on the air filter with 94444 m^3 of air collected from 2017/09/28 to 2017/10/04.

The radioactive ^{106}Ru content is estimated to be 250 TBq (specific activity of 1.22x10^{14} Bq/g, equals to 2 g)[5]. However, the total amount of Ru on the filter is measured with 2.03 kBq (equal to 16.6 pg). The estimated mass of ^{103}Ru released from the source is about 60 µg.

Supplementary Note 5. Russia's nuclear power reactor fleet

At the time of the incident, 35 nuclear power reactors were listed as active in the Russian Federation (Supplementary Table 8; [6]). The VVER-210 is the discontinued lower-power version of the VVER family, whereas VVER-440 is still frequently used. The related isotopic characteristics of both reactor types illustrate the great similarities within the early VVER families (Fig. 3). The reactors of the Russian power reactor fleet are operated with different grades of enriched uranium. Fuel for VVER-1000 reactors is enriched to 4.3 % ^{235}U; VVER-

440 fuel to 3.6 %; fuel for High Power Channel-type Reactors (RBMK) is enriched to 2.0 %; and the fast breeder reactor BN-600 fuel to 20 % [6].

According to Bolshov[7], spent nuclear fuel from VVER-440 and BN-600 reactors is reprocessed at the Production Association Mayak, whereas VVER-1100 fuel is sent for centralized wet storage at the Krasnoyarsk MCC (information as of 2007). Fuel from RBMK and EPG reactors is stored in the NPP repositories. Russia also possesses a significant inventory of spent nuclear fuel from its naval fleet, which is also processed at the Mayak facility[7].

Supplementary Figures

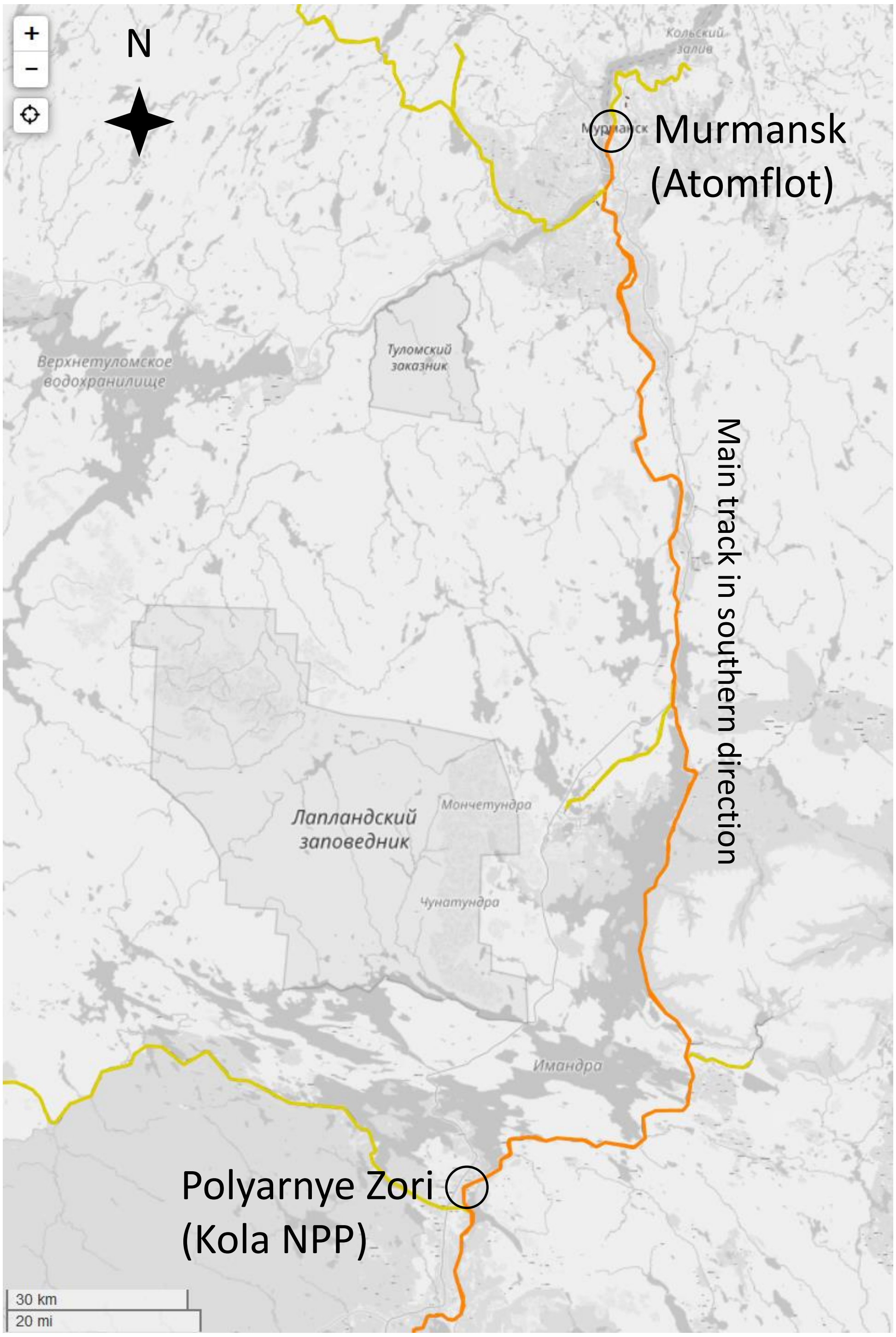

Supplementary Figure 1. Main railway connections on the Kola Peninsula that may be used to connect Murmansk in southern direction. Data © OpenStreetMap contributors (ODbL), map image OpenStreetMap/OpenRailwayMap (CC-BY-SA 2.0)[8].

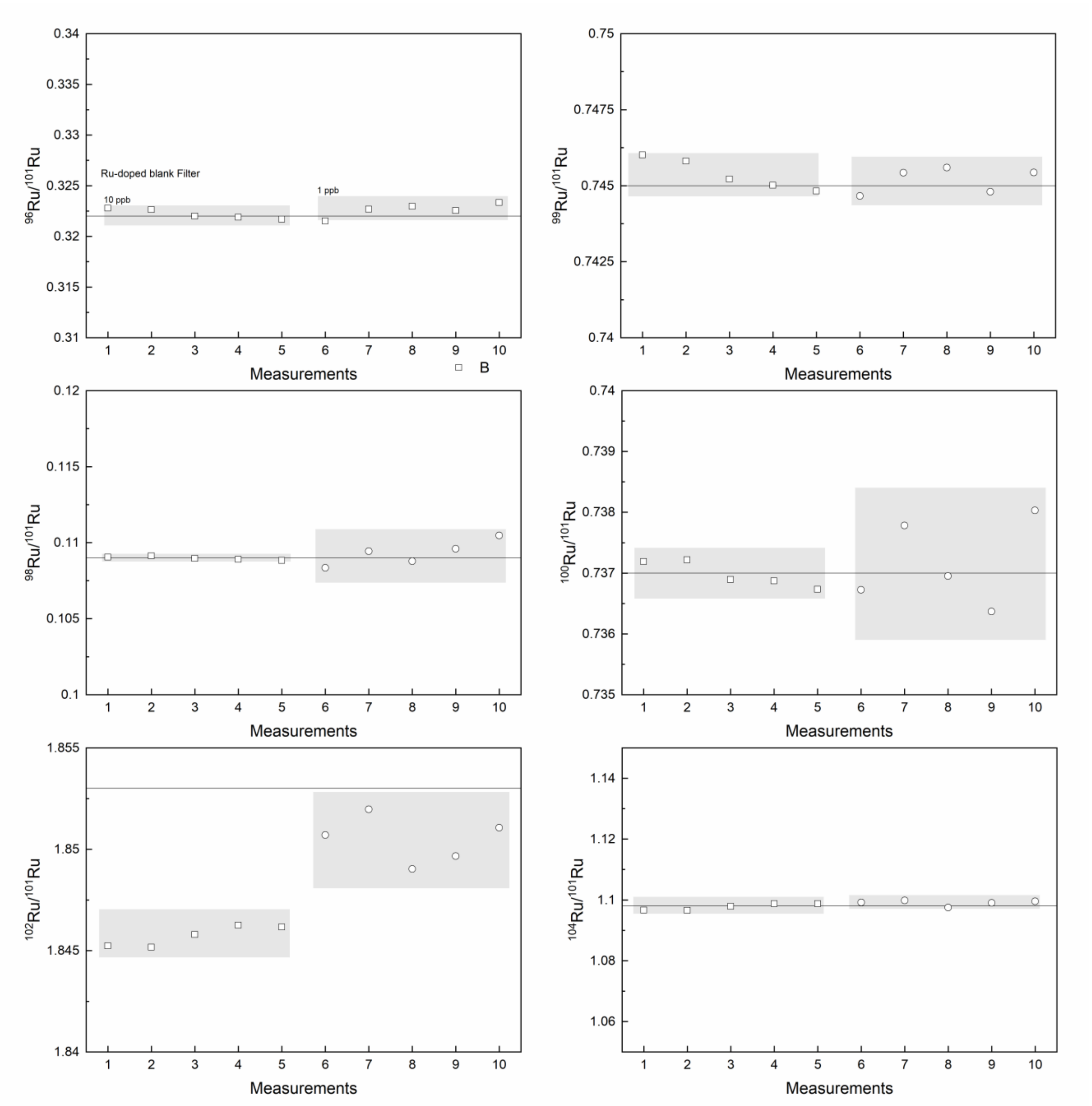

Supplementary Figure 2. Reproducibility and accuracy of Ru isotopic composition measurements of a Ru-doped air filters by MC-ICPMS. Gray boxes give the standard deviation (2 s.d.) on the determined isotope ratios for 10 ppb and 1 ppb solution measurements, respectively. The solid lines define the natural Ru isotope composition[9]. Deviation from the natural $^{102}Ru/^{101}Ru$ is produced by larger Pd interferences during the measurements of the 10 ppb solution.

Supplementary Tables

Supplementary Table 1. Air filter collection dates, passed air volume, weights of samples, and measured activity of ^{103}Ru and ^{106}Ru.

Start of collection	End of collection	Weight [g]	Passed air volume [m³]	^{103}Ru/^{106}Ru acitivity [Bq]
2015/07/19	2015/07/19	2.49909	20000	-
2017/09/27	2017/09/28	2.48189	20000	-
2017/09/28	2017/10/04	2.51576	94444	0.66/2030 (as of 2017/09/26)
2018/01/24	2018/01/25	2.53357	20000	-
2018/02/26	2018/02/27	2.46478	19099	-
Blank filter		2.4895	0	-

Supplementary Table 2. Ruthenium isotopic composition of the Alfa Aesar standard solutions and Ru-doped blank filter measured by MC-ICPMS. Instrumental mass bias was corrected by normalizing the bracketing standards to $^{99}Ru/^{101}Ru$ of 0.745 and using the average isotope fractionation factor (β) of the bracketing standards on the sample measurements (for details see Methods).

Sample	n	$^{96}Ru/^{101}Ru$	$^{98}Ru/^{101}Ru$	$^{99}Ru/^{101}Ru$	$^{100}Ru/^{101}Ru$	$^{102}Ru/^{101}Ru$	$^{104}Ru/^{101}Ru$
Alfa Aesar standard							
10 ppb (2 s.d.)	103	0.3220(3)	0.1090(2)	0.7451	0.7370(1)	1.8532(4)	1.0978(3)
1 ppb (2 s.d.)	74	0.321(2)	0.109(1)	0.7451	0.737(1)	1.854(2)	1.098(2)
Ru-doped blank Filter							
10 ppb		0.323	0.109	0.746	0.737	1.845	1.097
10 ppb		0.323	0.109	0.746	0.737	1.845	1.097
10 ppb		0.322	0.109	0.745	0.737	1.846	1.098
10 ppb		0.322	0.109	0.745	0.737	1.846	1.099
10 ppb		0.322	0.109	0.745	0.737	1.846	1.099
Average (2 s.d.)	5	0.322(1)	0.1090(2)	0.745(1)	0.7370(4)	1.846(1)	1.098(2)
1 ppb		0.322	0.108	0.745	0.737	1.851	1.099
1 ppb		0.323	0.109	0.745	0.738	1.852	1.100
1 ppb		0.323	0.109	0.746	0.737	1.849	1.097
1 ppb		0.323	0.110	0.745	0.736	1.850	1.099
1 ppb		0.323	0.110	0.745	0.738	1.851	1.100
Average (2 s.d.)	5	0.323(1)	0.109(2)	0.745(1)	0.737(1)	1.850(1)	1.099(2)

Supplementary Table 3. Ruthenium isotopic composition of air filters measured by MC-ICPMS. Instrumental mass bias was corrected by normalizing the bracketing standards to $^{99}Ru/^{101}Ru$ of 0.745 and using the average isotope fractionation factor (β) of the bracketing standards on the sample measurements (for details see Methods). Denoted errors (parentheses) are the standard error (2 s.e.) for n < 3 (measurements) or the standard deviation (2 s.d.) for n ≥ 3.

Sample	n	$^{96}Ru/^{101}Ru$	$^{98}Ru/^{101}Ru$	$^{99}Ru/^{101}Ru$	$^{100}Ru/^{101}Ru$	$^{102}Ru/^{101}Ru$	$^{104}Ru/^{101}Ru$
Reference filters							
2015/07/19-2015/07/20							
1 ppb		0.330	0.111	0.786	0.735	1.848	1.096
1 ppb		0.331	0.112	0.785	0.736	1.847	1.095
1 ppb		0.328	0.110	0.784	0.734	1.849	1.097
Average (2 s.d.)	3	0.330(3)	0.111(3)	0.785(2)	0.735(2)	1.848(1)	1.096(2)
2017/09/27-2017/09/28							
1ppb (2 s.e.)	1	0.3211(1)	0.1150(1)	0.762(1)	0.743(1)	1.848(1)	1.095(1)
2018/01/24-2018/01/25							
10 ppb		0.322	0.113	0.787	0.737	1.852	1.098
1 ppb		0.324	0.114	0.788	0.737	1.851	1.096
1 ppb		0.322	0.112	0.784	0.736	1.854	1.100
1 ppb		0.322	0.113	0.787	0.737	1.852	1.098
1 ppb		0.324	0.114	0.787	0.737	1.853	1.097
1 ppb		0.323	0.112	0.786	0.737	1.852	1.098
Average (2 s.d.)	6	0.323(2)	0.113(1)	0.787(3)	0.7369(8)	1.853(2)	1.098(3)
2018/02/26-2018/02/27							
10 ppb		0.325	0.113	0.801	0.736	1.849	1.096
1 ppb		0.324	0.111	0.802	0.735	1.847	1.095
1 ppb		0.324	0.111	0.802	0.735	1.848	1.096
Average (2 s.d.)	3	0.3244(4)	0.112(3)	0.802(1)	0.735(1)	1.848(2)	1.096(2)
Average all filters (2 s.d.)	4	0.325(8)	0.113(4)	0.784(32)	0.738(7)	1.849(4)	1.096(2)
Non-natural Ru air filter							
2017/09/28-2018/10/04							
10 ppb		0.0616(1)	0.02048(8)	0.1424(2)	0.2161(2)	1.1306(2)	0.6894(1)
1 ppb		0.0619(3)	0.0207(4)	0.1427(3)	0.2164(3)	1.1300(4)	0.6888(2)
Wdt. Average	2	0.0616(1)	0.0205(1)	0.1425(14)	0.2162(1)	1.131(3)	0.689(3)

Supplementary Table 4. Ruthenium isotopic compositions of civilian reactor types, low-burnup nuclear fuel, Hanford Site groundwater, and ^{235}U thermal fission yields.

Type of production	$^{96}Ru/^{101}Ru$	$^{98}Ru/^{101}Ru$	$^{99}Ru/^{101}Ru$	$^{100}Ru/^{101}Ru$	$^{102}Ru/^{101}Ru$	$^{104}Ru/^{101}Ru$
PWR[a]-MOX[b]	0	0	0	0.143	1.171	1.129
BWR[c]-MOX	0	0	0	0.137	1.155	1.109
PWR-UO_2[d]	0	0	0	0.188	1.08	0.775
BWR-UO_2	0	0	0	0.182	1.078	0.792
VVER[e] 440	0	0	0	0.108	0.999	0.682
VVER[e] 210	0	0	0	0.086	0.996	0.733
low-burnup nuclear fuel[f]	0	0	0	-	0.826	-
Hanford Site groundwater[g]	0	0	0	-	0.801(21)	-
^{235}U thermal fission[h]	0	0	0	-	0.829	-
^{239}Pu thermal fission[h]	0	0	0	-	0.984	-

[a]Pressurized water reactor, after [5], [b]Mixed oxide fuel, after [5], [c]Boiling water reactor, after [5], [d]Uranium dioxide fuel, after [5], [e]Water-water energetic reactor, after [5], [f]Average of values from[2], [g]Average of Hanford Site groundwater samples from [1], [h]Calculated or measured thermal neutron fission yields following[3].

Supplementary Table 5. Enrichment and burn up parameters for the simulated reactor types.[4]

Reactor type –Fuel type	Nuclide	U enrichment or Pu/U composition %	Burnup GWd/t heavy metal
PWR-MOX	Pu total	8.6	55
	Nat. U	91.4	
BWR-MOX	Pu total	6.3	50
	Nat. U	93.6	
PWR-UO_2	^{235}U	4.4	55
BWR-UO_2	^{235}U	3.5	50
VVER 440	^{235}U	3.6	30
VVER 210	^{235}U	2.0	20

Supplementary Table 6. Impurities in the fuel.[4]

Impurity	F	H_2O	B	Fe	Si	Ni	N	C	Cl	Ca	Ag	Bi	Co	Cu	Mg	Mo
mg/kg	4	2	0.5	15	6	3.5	30	4	4.5	10	5	5	5	5	5	5

Impurity	Na	Pb	Sn	V	Zn	Ti	Th	Ta	P	W	Li	Al	K	Cr	Mn
mg/kg	5	5	5	5	5	5	5	5	5	5	1	21	10	3	1

Supplementary Table 7. Ruthenium isotope abundances of air filter samples and fission-generated Ru from VVER reactors relative to natural Ru abundances used in Fig. 2.

Sample	^{96}Ru [%]	^{98}Ru [%]	^{99}Ru [%]	^{100}Ru [%]	^{101}Ru [%]	^{102}Ru [%]	^{104}Ru [%]
Natural Ru[a]	5.54(14)	1.87(3)	12.76(14)	12.60(7)	17.06(2)	31.55(14)	18.62(27)
Alfa Aesar standard	5.49(1)	1.859(3)	12.706(2)	12.567(2)	17.053(3)	31.603(5)	18.721(4)
Ru-doped blank filter	5.50(2)	1.861(4)	12.73(2)	12.583(8)	17.074(2)	31.51(1)	18.74(3)
Reference filters							
2015/07/19-2015/07/20	5.59(4)	1.88(5)	13.29(3)	12.45(2)	16.935(2)	31.30(5)	18.56(5)
2017/09/27-2017/09/28	5.46(1)	1.95(2)	12.96(1)	12.62(1)	16.99(1)	31.41(1)	18.61(1)
2018/01/24-2018/01/25	5.46(2)	1.91(2)	13.31(5)	12.47(1)	16.922(7)	31.35(4)	18.58(5)
2018/02/26-2018/02/27	5.483(1)	1.88(4)	13.55(3)	12.43(1)	16.90(2)	31.236(5)	18.52(2)
Average all filters (2 s.d.)	5.50(12)	1.91(7)	13.28(48)	12.49(18)	16.94(8)	31.32(15)	18.57(7)
Non-natural Ru air filter							
2017/09/28-2017/10/04	1.89(1)	0.63(1)	4.37(1)	6.63(1)	30.67(1)	34.67(2)	21.13(2)
VVER[b] reactors							
440	0	0	0	4.03	29.40	33.96	32.61
210	0	0	0	5.96	32.77	35.32	25.95

[a]Data from [9], [b]Water-water energetic reactor data calculated from [4].

Supplementary Table 8. Russia's power reactors (active in 2017), modified after[6].

Reactor/NPP	Type	MWe net, each	Commercial operation since	Licensed to, or scheduled closure
Balakovo 1	VVER-1000/320	988	5/86	2043
Balakovo 2	VVER-1000/320	988	1/88	2033
Balakovo 3	VVER-1000/320	988	4/89	2049
Balakovo 4	VVER-1000/320	988	12/93	2053
Beloyarsk 3	BN-600 FBR	560	11/81	2025
Beloyarsk 4	BN-800 FBR	789	10/16	2056
Bilibino 1-4	LWGR EGP-6	11×4	12/74-1/77	12/2021
Kalinin 1	VVER-1000/338	988	6/85	2045
Kalinin 2	VVER-1000/338	988	3/87	2047
Kalinin 3	VVER-1000/320	988	11/2005	2065
Kalinin 4	VVER-1000/320	988	9/2012	2072
Kola 1	VVER-440/230	432	12/73	2033
Kola 2	VVER-440/230	411	2/75	2029
Kola 3	VVER-440/213	440	12/82	2027
Kola 4	VVER-440/213	440	12/84	2039
Kursk 1	RBMK	971	10/77	2022
Kursk 2	RBMK	971	8/79	2024
Kursk 3	RBMK	971	3/84	2029
Kursk 4	RBMK	925	2/86	2031
Leningrad 1	RBMK	971	11/74	2018
Leningrad 2	RBMK	971	2/76	2021
Leningrad 3	RBMK	971	6/80	2025
Leningrad 4	RBMK	925	8/81	2026
Novovoronezh 4	VVER-440/179	385	3/73	2032
Novovoronezh 5	VVER-1000/187	950	2/81	2035
Novovoronezh II-1*	VVER-1200/392M	1114	2/2017	2077
Smolensk 1	RBMK	925	9/83	2028
Smolensk 2	RBMK	925	7/85	2030
Smolensk 3	RBMK	925	1/90	2050
Rostov 1	VVER-1000/320	990	3/2001	2030?
Rostov 2	VVER-1000/320	990	10/2010	2040
Rostov 3	VVER-1000/320	1011	9/2015	2045
**Total: 35**		**26,911 MWe**		

Supplementary References

1 Dresel, P. E., Evans, J. C. & Farmer, O. T. *Investigation of isotopic signatures for sources of groundwater contamination at the Hanford Site*, https://www.pnnl.gov/main/publications/external/technical_reports/pnnl-13763.pdf (2002).

2 Byerly, B. *et al.* Determination of initial fuel state and number of reactor shutdowns in archived low-burnup uranium targets. *J. Radioanal. Nucl. Chem.* **307**, 1871-1876 (2016).

3 Shima, M., Thode, H. G. & Tomlinson, R. H. Cumulative yields of stable and long-lived isotopes of ruthenium and palladium in neutron-induced fission. *Can. J. Phys.* **56**, 1340-1352 (1978).

4 Pfeiffer, F. *et al. Abfallspezifikation und Mengengerüst, GRS-278 (in German)*, https://www.grs.de/en/node/1749 (2011).

5 Masson, O. *et al.* Airborne concentrations and chemical considerations of radioactive ruthenium from an undeclared major nuclear release in 2017. *Proc. Natl. Acad. Sci. USA* **116**, 16750-16759 (2019).

6 World Nuclear Association. *Russia's nuclear fuel cycle*, https://www.world-nuclear.org/information-library/country-profiles/countries-o-s/russia-nuclear-fuel-cycle.aspx (2019).

7 Bolshov, L. *Safety for nuclear fuel cycle in the Russian Federation*, http://www.oecd-nea.org/nsd/workshops/fcsafety/proceedings/documents/Session_3_2_Presentation_4.pdf (2007).

8 *Open Railway Map*, https://www.openrailwaymap.org/ (2019).

9 Berglund, M. & Wieser, M. E. Isotopic compositions of the elements 2009 (IUPAC technical report). *Pure Appl. Chem.* **83**, 397-410 (2011).
